# Gut Microbial Dysbiosis Differs in Two Distinct Cachectic Tumor-Bearing Models Consuming the Same Diet

**DOI:** 10.3390/nu16071076

**Published:** 2024-04-06

**Authors:** Lauri O. Byerley, Brittany Lorenzen, Hsiao-Man Chang, William G. Hartman, Michael J. Keenan, Ryan Page, Meng Luo, Scot E. Dowd, Christopher M. Taylor

**Affiliations:** 1 Department of Physiology, School of Medicine, Louisiana State University Health Sciences Center, 1901 Perdido St., New Orleans, LA 70112, USAhchan2@lsuhsc.edu (H.-M.C.);; 2 School of Nutrition and Food Sciences, Louisiana State University, 297 Knapp Hall, Baton Rouge, LA 70803, USA; mkeenan@agcenter.lsu.edu (M.J.K.); rpage1@lsu.edu (R.P.); 3 Department of Microbiology, Immunology and Parasitology, School of Medicine, Louisiana State University Health Sciences Center, 1901 Perdido St., New Orleans, LA 70112, USA; mluo2@lsuhsc.edu (M.L.); ctay15@lsuhsc.edu (C.M.T.); 4 Molecular Research LP, 503 Clovis Rd., Shallowater, TX 79363, USA; sdowd@mrdnalab.com

**Keywords:** gut microbiome, walnuts, cancer, cachexia, weight loss

## Abstract

The impact of cancer cachexia on the colonic microbiota is poorly characterized. This study assessed the effect of two cachectic-producing tumor types on the gut microbiota to determine if a similar dysbiosis could be found. In addition, it was determined if a diet containing an immunonutrient-rich food (walnuts) known to promote the growth of probiotic bacteria in the colon could alter the dysbiosis and slow cachexia. Male Fisher 344 rats were randomly assigned to a semi-purified diet with or without walnuts. Then, within each diet group, rats were further assigned randomly to a treatment group: tumor-bearing ad libitum fed (TB), non-tumor-bearing ad libitum fed (NTB-AL), and non-tumor-bearing group pair-fed to the TB (NTB-PF). The TB group was implanted either with the Ward colon carcinoma or MCA-induced sarcoma, both transplantable tumor lines. Fecal samples were collected after the development of cachexia, and bacteria species were identified using 16S rRNA gene analysis. Both TB groups developed cachexia but had a differently altered gut microbiome. Beta diversity was unaffected by treatment (NTB-AL, TB, and NTB-PF) regardless of tumor type but was affected by diet. Also, diet consistently changed the relative abundance of several bacteria taxa, while treatment and tumor type did not. The control diet increased the abundance of *A. Anaeroplasma*, while the walnut diet increased the genus *Ruminococcus*. There were no common fecal bacterial changes characteristic of cachexia found. Diet consistently changed the gut microbiota, but these changes were insufficient to slow the progression of cachexia, suggesting cancer cachexia is more complex than a few gut microbiota shifts.

## 1. Introduction

Recent studies have demonstrated a synergistic relationship between the microbes in our gut and many physiological processes within our body [1,2]. Sometimes, those physiological processes go awry, resulting in tumor growth [3]. The effect of a tumor on our gut microbes has not been clearly established. The growth of a tumor is known to impact many physiological processes, including promoting the unexplained loss of body tissues or cachexia [4,5]. This study investigated the impact of tumor-driven cachexia on the gut microbiome and whether a diet rich in walnuts can alter the observed dysbiosis.

Cachexia occurs in many terminal disease states, such as cancer, heart disease, etc., and many studies have described the physiological processes perturbed by tumor-driven cachexia. More than 50% of cancer patients experience unexplained weight loss or cachexia [6], which can be devastating, affecting the patient’s quality and length of life as well as response to treatment [7]. Ultimately, preventing cancer cachexia would be best, but despite decades of research, there are no known cures. The etiology of cancer cachexia remains a mystery.

Genomic techniques have allowed us to identify the microbes in our environment, including those on and in our body. Our gastrointestinal tract contains thousands of bacterial species, with the largest population located in the colon. Many correlative studies have demonstrated a profound communication network between the colonic bacterial communities and the host’s cells [2,8]. For example, gut bacteria are critical for developing and training the baby’s immune system, which continues throughout one’s lifespan [9,10]. In response, the host’s immune system secretes molecules that target particular bacterial groups in the colon and regulate their growth [10,11]. Both the gut microbiome and the host’s immune system work in concert. The gut microbiota plays an essential role in maintaining the homeostasis of the host [2,10]. Bearing a tumor changes the host’s immune system and potentially its symbiotic relationship with the gut microbiota [3,12,13].

It has been proposed that gut dysbiosis is one of the factors that contribute to the development and progression of cancer cachexia. Dysbiosis can lead to increased intestinal permeability, impaired immune function, and chronic inflammation, all of which can affect the metabolism and muscle function of the host [14]. Many of these changes are hallmarks of cancer cachexia as well [15]. Several studies have shown that cancer cachexia is associated with changes in the composition and diversity of the gut microbiota, with a decrease in beneficial bacteria, such as *Ruminococcaceae* [15], *Lachnospiraceae* [15,16], and *Lactobacillus* [17], and an increase in harmful bacteria, such as *Bacteroidetes* [18], *Enterobacteriaceae* [16,18,19], and *Parabacteroides* [18]. These changes may influence the production of metabolites, cytokines, and hormones that modulate appetite, energy expenditure, and muscle and fat mass [20].

Most studies to date are limited to murine models with colon cancer, neuroblastoma, or leukemia. *Lactobacillus reuteri* and *Lactobacillus gasseri* were low in cachectic mice with leukemia [17]. Potgens et al. linked cachexia, induced by colon carcinoma 26, with *Klebsiella oxytoca*, a specific gut bacterial species that altered gut barrier function in cachectic mice with colon carcinoma [15]. A particular strain, *Faecalibacterium prausnitzii* A2–165 (DSM 17677), was unsuccessful at reversing cancer cachexia in the same mouse model [21]. One study in cachectic human cancer patients found an unknown genus from the *Enterobacteriaceae* family (*p* < 0.01) and that Proteobacteria (*p* < 0.001) and *Veillonella* (*p* < 0.001) were more abundant [22]. Thus, a clear pattern of gut microbiota dysbiosis has not emerged.

The primary goal of this study was to determine if tumor-driven cachexia produces a typical pattern of dysbiosis. While changes in a few individual gut microbes have been reported, no consistent pattern of changes has been identified. This study used two transplantable tumor models, carcinoma and sarcoma, to determine if cachexia driven by different tumor types is associated with similar changes in colonic microbiota’s relative abundance. The carcinoma we selected grows more slowly than the sarcoma, but both models have been used extensively to study cancer cachexia. In addition, rats were selected since previous studies have used mice models. Using a rodent model allows the investigator to control extraneous microbiota influencers, such as diet. 

In humans, daily lifestyle choices, such as diet, sleep, and physical activity, are known to change the relative abundance of bacteria within the colon [23]. Since diet can alter the gut microbiota, it may be one way to influence the tumor’s effect on the host’s gut microbiota and the cachectic process. Thus, the second goal of our study was to determine whether diet could remediate the gut dysbiosis observed in cachectic tumor-bearing rats. Walnuts were selected to add to the diet because they have been shown to promote probiotic gut bacteria in non-tumor-bearing rats [24]. Also, walnuts are an excellent source of two dietary constituents with known anti-cachectic properties [25,26]: omega-3 fatty acid (particularly α -linolenic acid) and antioxidants. Finally, several studies have shown that walnuts can slow or prevent breast and prostate tumor growth in genetically programmed mice [25] and xenografts [26]. For the studies reported here, walnuts were added to the animal’s diet without compromising nutritional quality.

## 2. Materials and Methods

### 2.1. Study Design

**Figure 1:**
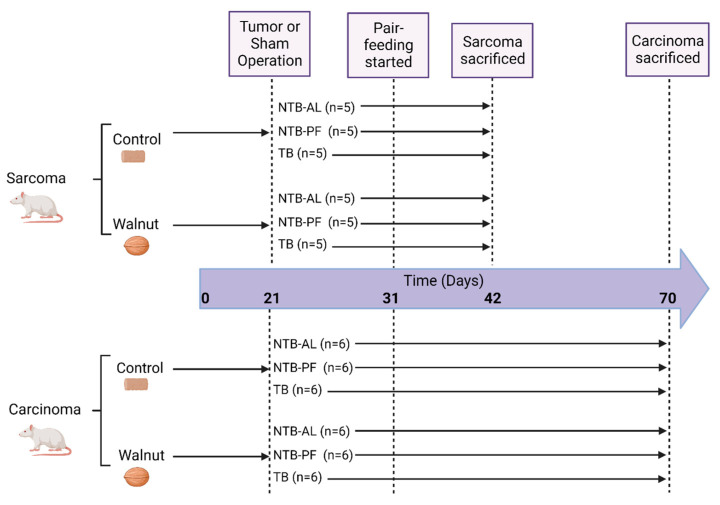
Study design and timeline (created with BioRender.com).

This study was approved by the Institutional Care and Use Committee at the Louisiana State University Health Sciences Center (LSUHSC) in New Orleans, LA, USA. One cohort of animals consisted of thirty male Fischer 344 rats, and the second cohort was thirty-six male Fischer 344 rats. The animals were housed in the LSUHSC vivarium under controlled conditions, constant temperature, and a 12 h light/dark cycle. The animals were maintained on rat chow for one week, and at the end of the week, they were weighed and randomly assigned to one of two diet groups: (1) control and (2) walnut. Each animal was singly housed and fed their assigned diet for the remainder of the study. The animals were allowed to adjust to their single housing and diet for three weeks before tumor implantation. The study’s design is shown in Figure 1; the diets are described under Section 2.2.

The day before tumor implantation, the animals were weighed and randomly assigned to one of three treatment groups: (1) a tumor-bearing (TB) group that was implanted with the tumor and fed ad libitum; (2) a non-tumor-bearing (NTB-AL) group that was sham-operated and fed ad libitum; and, (3) a pair-fed (NTB-PF) group that was sham-operated and given the amount of food the TB animals ate the previous 24 h. This grouping is referred to as treatment (NTB-AL, TB, and NTB-PF). NTB-PF animals were assigned by weight to a TB animal, so there was no more than a two-gram weight difference between the TB and NTB-PF animals. The NTB-AL animals were also weight-matched (±5 g) to NTB-PF and TB animals. 

After assignment to their treatment group, each animal was anesthetized using isoflurane. A 2 × 2 × 2 mm chunk of the Ward colon carcinoma (carcinoma) or MCA-induced sarcoma (sarcoma), referred to as tumor type, was obtained from a donor tumor-bearing animal and implanted subcutaneously on the left hind flank. Cells for the Ward colon carcinoma tumor line were graciously supplied by Dr. Vickie Baracos at the University of Alberta, Canada. The MCA sarcoma cells were obtained from Dr. Lauri Byerley’s laboratory. NTB-PF and NTB-AL animals received the same operation (sham) as the TB animals but did not receive the tumor cells. 

Animals were weighed and fed daily for 21 days (sarcoma) or 49 days (carcinoma) and then euthanized. Twelve hours prior to euthanasia, food was removed from the animals’ cages to ensure they were in a similar metabolic state. At euthanasia, the animal was anesthetized using isoflurane, blood was collected by cardiac puncture, and the abdominal artery was cut to ensure death. Fecal samples were collected aseptically from the descending colon, frozen in liquid nitrogen, and stored at −80° C until DNA isolation.

### 2.2. Diets

The diet was reported previously and identical to the one used by Hardman et al. [25]. Briefly, the diet was based on the AIN-76 diet. The protein (walnut: 15.6 g/100 g; control: 15.5 g/100 g), fat (walnut: 4.3 g/100 g, control: 5.8 g/100 g), carbohydrate (walnut: 61.7 g/100 g; control: 60.9 g/100 g), and crude fiber (walnut: 3.67 g/100 g; control: 2.7 g/100 g) were adjusted in the control diet for the walnuts, so both had a similar macronutrient composition. 

Each diet was made in small batches. The walnuts were ground to a fine state and mixed with the other ingredients. When the diet was the consistency of cookie dough, it was rolled, vacuum-sealed in small batches, and frozen at −20 °C until fed to the animals. The diet was thawed at the time of feeding, and a weighed cube was given to the animal. A fresh diet was provided every two days. Pair-feeding started ten days after surgery to allow the animals to recover from the surgery. Previous studies by our group have shown that food intake between TB and NTB-AL animals was not different until ten days after tumor implant. On the eleventh day after tumor implantation, the NTB-PF received the amount of food their matched TB animals consumed 24 h earlier.

### 2.3. DNA Isolation and PCR Amplification

A protocol developed by the LSUHSC School of Medicine Microbial Genomics Resource Group (http://metagenomics.lsuhsc.edu/mgrg (access on 1 January 2024)) was used to extract total DNA from approximately 0.25 g of feces. This method has been previously published [17]. The QIAamp DNA Stool Kit (Qiagen, Germantown, MD, USA) was modified to include bead-beating and RNAase treatment steps. 

### 2.4. Sequencing 

The procedure was previously published [17]. Briefly, the 16S rRNA gene (V3-V4 hypervariable region) was PCR amplified using V3F = CCTACGGGAGGCAGCAG and V4R = GGACTACHVGGGTWTCTAAT primers, Illumina adaptors, and molecular barcodes [18]. Each sample was ligated with Illumina indexes and multiplexed for sequencing on a single Illumina MiSeq run using the Illumina V3 600-cycle sequencing kit (Illumina, San Diego, CA, USA) in paired-end mode. Microbial Mock Community HM-276D (BEI Resources, Manassas, VA, USA) was used as a positive control.

### 2.5. Quality Filtering/Picking

Forward read files were processed through the UPARSE pipeline [19]. Reverse reads were discarded due to persistent read quality issues with the reverse sequencing reads from Illumina V3 sequencing kits. Reads were truncated to a uniform length of 280 bp and reads with quality scores less than 16 were filtered out. The UPARSE pipeline steps described by Edgar [27] were performed in sequence, and OTU clusters were formed at 97% with chimeric OTUs removed from the data. After quality filtering, reads were analyzed using QIIME 1.9.0 (Quantitative Insights Into Microbial Ecology) with the DADA2 plugin [20]. Forward and reverse reads were truncated to a uniform length of 240 bp, and 20 bp were trimmed off the front of each read to remove the primer. DADA2-identified amplicon sequence variants (ASVs) were merged, and any that ranged outside the expected 250–255 bp amplicon length were discarded. Any ASVs that appeared in only one sample were removed using contingency-based filtering, and chimeric ASVs were removed using the consensus method. ASVs were aligned using MAFFT [28] and FastTree [29], and a phylogenetic tree for diversity analysis was built. Greengenes v13.8 was used for taxonomic classification [30]. After primary data analysis, the remaining reads were analyzed using QIIME2 [31].

### 2.6. Microbial Community Analysis

Sixty-six samples (30 sarcoma and 36 carcinoma) were included in the QIIME analysis with read counts ranging from 11,619 to 147,455 with an average read count per sample of 91,143 (sarcoma) and 92,036 (carcinoma). Alpha rarefaction was performed at a level of 11,619 reads to include all samples. Alpha rarefaction plots were produced by plotting the number of sequences in a sample against several different diversity metrics, for example, Shannon, Simpson, and Chao1. Beta diversity was determined by principal coordinate analysis using both unweighted and weighted UniFrac metrics. Emperor 3D viewer was used to visualize the plots [32,33]. 

### 2.7. Predicted Functional Pathways

Potential microbial functions were identified from the 16S sequencing data. The raw data were formatted and imported into QIIME2. Closed-reference clustering against the Greengenes 13_5 97% OTUs reference database was used to develop a de-replicated feature table and representative sequences. The closed-reference OTU table was used as input into the PICRUSt [22] pipeline, and the resulting PICRUSt metagenome data were further analyzed by using STAMP (Statistical Analysis of Metagenomic Profiles) [23]. Pathways were labeled at Level 2 since several pathways were not classified at Level 1. From this data, KEGG (Kyoto Encyclopedia of Genes and Genomes) pathways were compared between NTB-AL, TB, and NTB-PF groups within each tumor type.

### 2.8. Statistical Analysis

Data are expressed as mean ± standard error of the mean (SEM). SAS (https://www.sas.com/en_us/home.html (access on 1 January 2024), SAS Institute Inc., Cary, NC, USA), SPSS (https://www.ibm.com/products/spss-statistics (access on 1 January 2024), IBM Corp. Released 2020. IBM SPSS Statistics for Windows, Version 27.0. IBM Corp: Armonk, NY, USA), and R (https://www.r-project.org/ (access on 1 January 2024), R Statistical Software (v4.1.2; R Core Team 2021)) software were used to analyze data statistically. Descriptive data such as mean and SEM were determined using SPSS. A *p*-value less than 0.05 was considered significant.

LEFSE was used to select the bacterial species to determine statistical differences between the groups to reduce the number of comparisons [34]. Differences among the two diet groups (control and walnut) and three treatment groups (NTB-AL, TB, and NTB-PF) for the selected bacterial species were determined using a two-way analysis of variance (SAS). Since multiple analyses were run, the Benjamini–Hochberg procedure was used to control for the false discovery rate. Briefly, the *p*-values were put in order from the smallest to largest, were ranked (rank of *i* = 1, *i* = 2, etc.), and a critical value (CV) was calculated as (*i*/*m*)*Q*, where *m* is the total number of tests and *Q* is the false discovery rate (0.05). Those taxa with a *p*-value less than the CV (*P* < ((*i*/m)*Q*)) were considered significant. If there was a significant effect, differences among the groups were determined using the Newman–Keuls. All taxa that were selected by LEFSE and their Benjamini–Hochberg values are shown in Table A1 and Table A2.

STAMP was used to determine statistical differences in functional pathways between the groups and generate post hoc (Tukey–Kramer) plots for each KEGG pathway significantly different between NTB-AL, TB, and NTB-PF animals. Bonferroni was used to correct for multiple analyses. Figures were created using GraphPad Prism v10 (https://www.graphpad.com/ (access on 1 January 2024), GraphPad Software, San Diego, CA, USA) and BioRender 2023 (https://www.biorender.com/ (access on 1 January 2024)). 

## 3. Results

**Figure 2:**
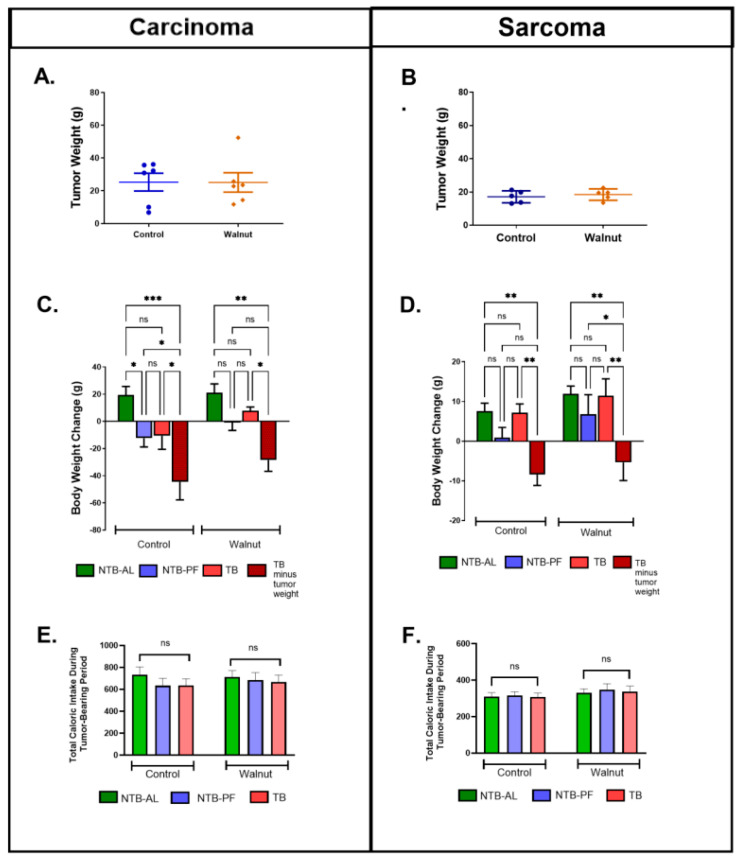
Tumor weight (**A**,**B**), weight change (difference from tumor implant or sham operation to euthanasia) (**C**,**D**), and total caloric intake (from implant to euthanasia) (**E**,**F**) for the NTB-AL, TB, and NTB-PF animals of both tumor types. Error bars represent mean ± SEM. (* *p* < 0.05, ** *p* < 0.01, *** *p* < 0.001, and ns = not significant).

Body weight did not differ significantly among the NTB-AL, TB, and NTB-PF groups (both tumor types) before the tumor or sham operation occurred (all: 330 ± 2; walnut: 330 ± 3; control: 330 ± 3). At the time of euthanasia, tumor weight was not significantly different between the control and walnut TB groups (both tumor types, Figure 2A,B). Host body weight (body weight minus tumor weight) at the time of euthanasia is shown in Figure 2C,D. At the time of euthanasia, host weight (total body weight minus tumor weight) for both the sarcoma- and carcinoma-bearing animals were significantly less than their matched NTB-AL animal regardless of diet, indicating they were cachectic. Total caloric intake (from implant to euthanasia) was not significantly altered between the walnut and control diets regardless of treatment (NTB-AL, TB, and NTB-PF) for either tumor type (Figure 2E,F). 

**Figure 3:**
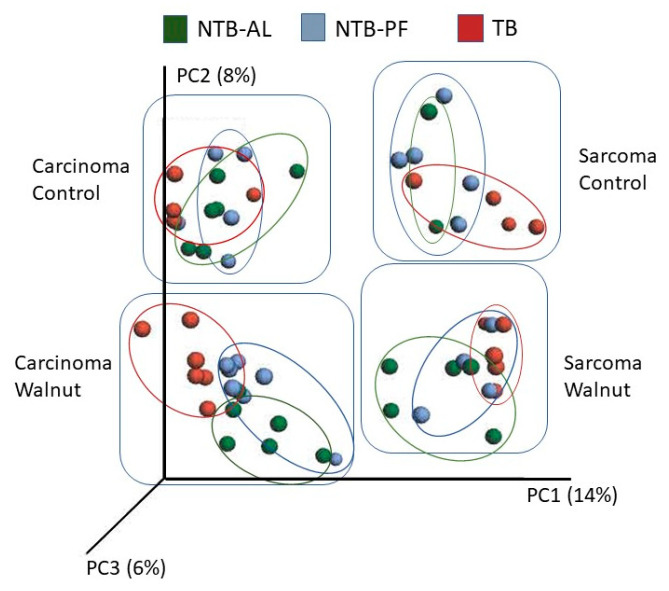
Beta diversity was measured by principal coordinate analysis (PCoA). Diet (walnut vs. control) and tumor type (sarcoma vs. carcinoma) promoted community separation. There was no clear community separation based on treatment (NTB-AL, TB, and NTB-PF) except for the carcinoma walnut group, where the TB animals were distinct from the NTB-AL and NTB-PF animals.

No differences in alpha diversity (within community diversity) using several different measures (Simpson, Shannon, Chao, observed taxa, and phylogenetic diversity) were found. Beta microbial diversity (differences between communities) is shown in Figure 3, and both diet and tumor type altered diversity. The walnut and control diets were clearly different from each other for both the sarcoma and carcinoma tumor types. However, the NTB-AL, TB, and NTB-PF overlapped within these four communities, so no differences could be determined except for the carcinoma walnut TB group, which differed from the carcinoma walnut NTB-PF and NTB-AL.

**Figure 4:**
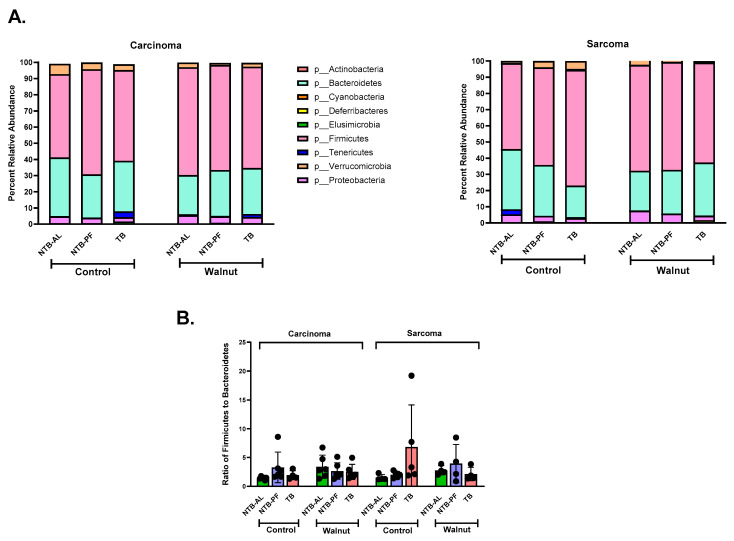
(**A**) Phyla abundance comparison between the carcinoma and sarcoma-bearing rats divided by diet and treatment. (**B**) Ratio of the relative abundance of Firmicutes to Bacteroidetes for both tumor types, diet, and treatment. No significant differences were observed. Error bars represent mean ± SEM.

Figure 4A shows nine phyla for the carcinoma and sarcoma animals on each diet and treatment. Together, Firmicutes and Bacteroidetes phyla comprised approximately 90% of the colonic microbiota, with 61% of the microbes from the Firmicutes phylum. The walnut diet consistently produced a similar relative abundance for the Firmicutes phyla (carcinoma: 67 ± 5% (NTB-AL), 63 ± 4% (TB), and 65 ± 4% (NTB-PF); sarcoma: 65 ± 4% (NTB-AL), 62 ± 5% (TB), and 67 ± 9% (NTB-PF)) regardless of tumor type and treatment (NTB-AL, TB, and NTB-PF). That was not the case for the control diet. The relative abundance of the Firmicutes phylum was lower in the NTB-AL animals for both tumor types (carcinoma: 52 ± 2%; sarcoma: 53 ± 2%), but higher in the sarcoma TB (carcinoma: 56 ± 4%; sarcoma: 72 ± 12%), and more in the carcinoma NTB-PF (carcinoma: 65 ± 2%; sarcoma: 60 ± 3%). The sarcoma-TB had a higher relative abundance compared to the carcinoma TB. The same pattern was observed for Bacteroidetes, but there was less variability in the relative abundance of the animals. 

The Firmicutes-to-Bacteroidetes (F/B) ratio has been proposed as a marker of gut dysbiosis. The Firmicutes-to-Bacteroidetes ratio from our study was not significantly different between treatment and diet for either tumor type (Figure 4B). Overall, Firmicutes dominated the OTU-level diversity by approximately 3-fold over the Bacteroidetes. The sarcoma TB consuming the control diet had the most variability and highest ratio. This group also had the lowest relative abundance of Bacteroidetes.

We also looked at the relative abundance of all species present in the stool sample. Several microbes were different for either the carcinoma or sarcoma treatment. Not corrected for multiple comparisons, these are shown in Table A1 and Table A2. We aimed to identify specific bacteria that were consistently elevated or reduced for both tumor types and, for this, corrected for multiple comparisons. Treatment did not significantly and consistently affect the relative abundance of any microbes. Only diet consistently and significantly altered the relative abundance of a few microbes shown in Table 1. For both tumor types, microbes from the Tenericutes phylum, order Anaeroplasmatales, had a significantly higher relative abundance in animals consuming the control diet regardless of treatment. The Tenericutes phylum’s relative abundance was low (0.84 ± 0.25%) compared to the Firmicutes and Bacerodiodetes phyla. The walnut diet significantly increased the relative abundance of several microbes from the Firmicutes phyla, particularly the Bacilli and Clostridia classes. 

**Figure 5:**
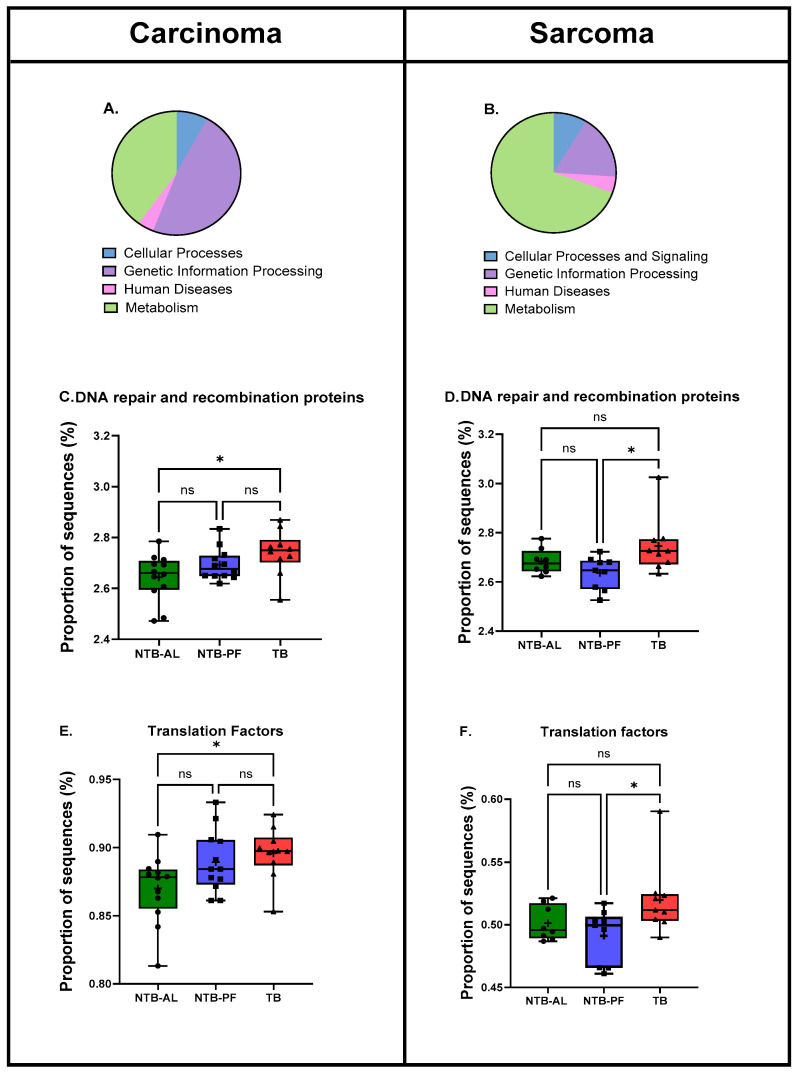
Predicted functional pathways. (**A**,**B**) show the percent contribution of the four most prominent Level 1 KEGG pathways in the two tumor types. Two pathways from Level 2 genetic information processing differed significantly among the treatment groups at KEGG Level 3 (**C**–**F**). (* *p* < 0.05, ns = not significant).

Differences in four functional pathways (KEGG Level 2) were predicted from the gene data (Figure 5A,B): cellular processing, genetic information processing, human diseases, and metabolism. Only genetic information processing had significantly different pathways at Level 3: DNA repair and recombination proteins and translation factors. No other pathways at Level 3 were significantly different. The same pathways were affected within each tumor type.

From 16s RNA, metabolic pathways that are up- or down-regulated can be predicted. KEGG is a hierarchical collection of pathway maps. Metabolism is one of these, which has seven broad categories. At Level 2, four predicted metabolic pathways emerged as different in the two tumor types. Their percentage difference is shown in Figure 5A,B. Each tumor type had a different percentage. At Level 3, we found two predicted metabolic pathways altered in carcinoma and sarcoma-bearing rats (Figure 5C–F). Pathways in DNA repair and recombination proteins and translation factors were elevated in the TB rats compared to the NTB-AL or the NTB-PF.

## 4. Discussion

Cancer cachexia, which is characterized by weight loss, muscle wasting, anorexia, and systemic inflammation, is a complex syndrome that affects many patients with advanced cancer [36]. These symptoms impair the quality of life, response to treatment, and the survival of cancer patients [37]. One of the factors that may contribute to the development and progression of cancer cachexia is the alteration of the gut microbiota [20]. The gut microbiota plays a vital role in maintaining the homeostasis of the host, but various factors, such as diet, infection, medication, or cancer itself, can promote dysbiosis [23].

This study used two distinctly different tumor types: carcinoma and sarcoma. Carcinomas account for 80 to 90% of all human cancers, while sarcomas are rare (<1% of adult human tumors). Each arises from different tissue types. Animal models for both tumor types have been developed, and the Ward colon carcinoma and the MCA-induced sarcoma have been used extensively to study cancer cachexia, so these were selected to compare their gut microbiota. The MCA-induced sarcoma is faster growing than the Ward colon carcinoma, but both produce cachexia unrelated to a reduced food intake. 

Diet has been studied extensively as a tool to improve the health and well-being of cachectic cancer patients. Several nutritional therapies (including prebiotics and probiotics) that target the gut microbiota have been tried in the last several decades. For example, Bindels et al. [17] administered lactobacilli to cachectic leukemia mice and found it decreased muscle atrophy. This same observation was confirmed in the colon carcinoma 26 mouse model that also develops cachexia [38]. We showed that walnuts increase probiotic bacteria, Lactobacillus, Ruminococcaceae, and *g. Roseburia* [24], so we, therefore, investigated if adding walnuts to the diet could improve the cachectic condition. We reported earlier that a diet with walnuts added does not slow muscle atrophy [39].

Several studies have shown that gut microbiota diversity and composition are altered in cachectic tumor-bearing animals and humans, thus supporting the notion that dysbiosis may be involved in the pathogenesis of this syndrome. Colonic dysbiosis has been reported in tumor-bearing mice, but the dysbiosis has not been compared to two distinctly different tumor types in a different species, rat. Alpha diversity represents a single sample’s richness and community diversity, such as the tumor-bearing animals. There are a variety of different measures that can be used to compare the richness and diversity between samples. Published results for these measures in cachectic tumor-bearing mice and humans are inconsistent. Jeong et al. [40] found that cachectic mice bearing Lewis lung cancer cell allografts had lower alpha diversity than non-tumor-bearing mice. We found neither community richness nor diversity was different regardless of tumor type (sarcoma vs. carcinoma), the diet consumed (walnut vs. control), and treatment (NTB-AL, TB, and NTB-PF). Ni et al. [41] also found no differences in alpha diversity in cachectic lung cancer patients compared to non-cachectic lung cancer patients. 

Beta diversity analysis quantifies the similarity or dissimilarity between microbiome pairs between samples, such as the walnut and control diets. Jeong et al. [40] found that cachectic mice bearing Lewis lung cancer cell allografts had distinct beta diversity compared to the non-tumor-bearing mice. We found a noticeable difference in beta diversity; tumor type and diet caused a significant separation in the composition of the gut microbiome, while treatment had no effect. These results suggest that tumor type and diet have a greater influence on beta diversity than bearing a tumor and developing cachexia.

While we did not find significant shifts in alpha diversity or the F/B ratio (a marker of gut dysbiosis), we observed a few changes in specific bacterial species. For this study, we corrected for multiple comparisons, which drastically reduced the number of significant species. Only diet significantly reduced or increased the relative abundance of several microbes for both tumor types. Diet is known to change the relative abundance of gut microbial communities. The control diet significantly increased the presence of two genera from the Anaeroplasmatales order for both the sarcoma and carcinoma animals. *Anaeroplasma* is an obligate anaerobe and resides in the gut at relatively low levels. There is minimal information on *Anaeroplasma* in human diseases, but it has been observed in an aging mouse model [42]. It is a member of the Tenericutes phylum, which has a low relative abundance compared to other members of the phylum level. De Maria, Y et al. [19] characterized the gut microbiome of mice bearing Lewis lung carcinoma. They found dysbiosis-involved representatives from seven phyla (Proteobacteria, Cyanobacteria, TM7, Actinobacteria, Bacteroidetes, Firmicutes, and Tenericutes), demonstrating a complex pattern. For the Tenericutes phylum, the F16 order was expanded, not Anaeroplamatales.

For the walnut diet, the class Bacilli and genus *Ruminococcus* had a significantly higher relative abundance for both tumor types. Bacilli are Gram-positive and often rod-shaped bacteria, widely distributed in nature, particularly soil. This class contains several well-known pathogens, including the bacteria that cause anthrax and *B. cereus*, a known food pathogen [43]. Although a relatively minor proportion of the gut microbiome, Bacilli class bacteria secrete a wide range of compounds [43]. *Ruminococcus* are butyrate-forming anaerobic Gram-positive bacteria that degrade and convert complex polysaccharides, like cellulose, into various nutrients, like glucose, for their hosts [44]. Byerley et al. reported that a walnut-rich diet increased the relative abundance of this bacteria in healthy, non-tumor-bearing rats [24]. Several other studies have reported increased [45] and decreased [46] *Ruminococcus* when walnuts are added to the human diet. We are unaware of any studies of cachectic animals or humans that have reported an increase in this particular bacterium. 

Ni et al. [41] used shotgun metagenomics to interrogate the gut microbiome of cachectic lung cancer patients. They reported that the catabolic pathways of certain complex carbohydrates and sugar derivatives and the anabolic pathways for several amino acid groups were significantly lower, while the polysaccharide pathways were enriched in the cachectic patients. Our 16s RNA analysis identified two pathways from the KEGG Level 2 genetic information processing pathway that significantly differed in both the sarcoma and carcinoma groups. These two pathways were related to DNA repair and translation factors. 

## 5. Conclusions

In summary, we found that cachexia, as a result of bearing a tumor, perturbed the gut microbiome, but the changes were not consistent across the two distinctly different tumor models examined. Therefore, we did not find a unique gut microbiome dysbiosis pattern that could be associated with cachexia. This suggests that gut microbiota changes are a consequence of cachexia and are unique to tumor type. Diet consistently altered the gut microbiome in both tumor types, but it was not enough to slow the progression of cachexia.

## Figures and Tables

**Table 1:** Microbes that were significantly different by diet.

Tumor Type	Dependent Variable	Pr > ChiSq	Rank	m =Number of Dependent Variables	CV for BH False Detect Rate ^1^
	**Higher in the control diet group**				
Sarcoma	p__Tenericutes|c__Mollicutes|o__Anaeroplasmatales	0.0023	5	20	0.013
Sarcoma	p__Tenericutes|c__Mollicutes|o__Anaeroplasmatales|f__Anaeroplasmataceae|g__Anaeroplasma	0.0023	5	20	0.013
Sarcoma	p__Tenericutes|c__Mollicutes|o__Anaeroplasmatales|f__Anaeroplasmataceae|g__Anaeroplasma|s__	0.0023	5	20	0.013
Carcinoma	p__Tenericutes|c__Mollicutes|o__Anaeroplasmatales	0.0033	22	45	0.0244
Carcinoma	p__Tenericutes|c__Mollicutes|o__Anaeroplasmatales|f__Anaeroplasmataceae|g__Anaeroplasma	0.0033	22	45	0.0244
Carcinoma	p__Tenericutes|c__Mollicutes|o__Anaeroplasmatales|f__Anaeroplasmataceae|g__Anaeroplasma|s__	0.0033	22	45	0.0244
	**Higher in the walnut diet group**				
Sarcoma	p__Firmicutes|c__Bacilli	0.0020	4	20	0.010
Sarcoma	p__Firmicutes|c__Clostridia|o__Clostridiales|f__Ruminococcaceae|g__Ruminococcus|Other	0.0006	2	20	0.005
Carcinoma	p__Firmicutes|c__Bacilli	0.0183	45	45	0.0500
Carcinoma	p__Firmicutes|c__Clostridia|o__Clostridiales|f__Ruminococcaceae|g__Ruminococcus|Other	0.0001	1	45	0.0011

^1^ Critical value for the Benjamini–Hochberg (BH) test as described by Benjamini and Hochberg [35]. Pr > ChiSq as determined by two-way ANOVA. False discovery rate is 0.05. All species observed for the sarcoma and carcinoma tumor types that ranked above the highest *p*-value but less than the critical value (CV) are shown.

## Data Availability

The data presented in this study are available on request from the corresponding author.

## Appendix A

**Table A1:** Significantly different taxa for diet.

Tumor Type	Dependent Variable	Pr > ChiSq from Kruskal–Wallis Test	Rank	m = Number of Dependent Variables	CV for BH False Detect Rate ^1^
	**Higher in the Control group**				
Sarcoma	p__Bacteroidetes|c__Bacteroidia|o__Bacteroidales|f__Bacteroidaceae|g__Bacteroides|Other	0.0024	7	20	0.018
Sarcoma	p__Bacteroidetes|c__Bacteroidia|o__Bacteroidales|f__Bacteroidaceae|g__Bacteroides|s__ovatus	0.0001	1	20	0.003
Sarcoma	p__Bacteroidetes|c__Bacteroidia|o__Bacteroidales|f__[Barnesiellaceae]	0.0179	11	20	0.028
Sarcoma	p__Bacteroidetes|c__Bacteroidia|o__Bacteroidales|f__Prevotellaceae|g__Prevotella|s__	0.0008	3	20	0.008
Sarcoma	p__Firmicutes|c__Bacilli|o__Lactobacillales|f__Lactobacillaceae|g__Lactobacillus|s__reuteri	0.0135	10	20	0.025
**Sarcoma**	**p__Tenericutes|c__Mollicutes|o__Anaeroplasmatales**	**0.0023**	**5**	**20**	**0.013**
**Sarcoma**	**p__Tenericutes|c__Mollicutes|o__Anaeroplasmatales|f__Anaeroplasmataceae**	**0.0023**	**5**	**20**	**0.013**
**Sarcoma**	**p__Tenericutes|c__Mollicutes|o__Anaeroplasmatales|f__Anaeroplasmataceae|g__Anaeroplasma**	**0.0023**	**5**	**20**	**0.013**
**Sarcoma**	**p__Tenericutes|c__Mollicutes|o__Anaeroplasmatales|f__Anaeroplasmataceae|g__Anaeroplasma|s__**	**0.0023**	**5**	**20**	**0.013**
Carcinoma	p__Actinobacteria|c__Actinobacteria|o__Actinomycetales	0.0025	16	45	0.0178
Carcinoma	p__Actinobacteria|c__Actinobacteria|o__Actinomycetales|f__MicrocoControlaceae	0.0031	17	45	0.0189
Carcinoma	p__Actinobacteria|c__Actinobacteria|o__Actinomycetales|f__MicrocoControlaceae|Other	0.0031	17	45	0.0189
Carcinoma	p__Actinobacteria|c__Actinobacteria|o__Actinomycetales|f__MicrocoControlaceae|Other|Other	0.0031	17	45	0.0189
Carcinoma	p__Bacteroidetes|c__Bacteroidia|o__Bacteroidales|f__Bacteroidaceae	0.0032	20	45	0.0222
Carcinoma	p__Bacteroidetes|c__Bacteroidia|o__Bacteroidales|f__Bacteroidaceae|g__Bacteroides	0.0032	20	45	0.0222
Carcinoma	p__Bacteroidetes|c__Bacteroidia|o__Bacteroidales|f__Bacteroidaceae|g__Bacteroides|s__	0.0152	43	45	0.0478
Carcinoma	p__Firmicutes|c__Bacilli|o__Lactobacillales|f__Carnobacteriaceae	0.0066	34	45	0.0378
Carcinoma	p__Firmicutes|c__Bacilli|o__Lactobacillales|f__Carnobacteriaceae|g__Granulicatella	0.0066	34	45	0.0378
Carcinoma	p__Firmicutes|c__Bacilli|o__Lactobacillales|f__Carnobacteriaceae|g__Granulicatella|s__	0.0066	34	45	0.0378
Carcinoma	p__Firmicutes|c__Clostridia|o__Clostridiales|f__Ruminococcaceae|g__Ruminococcus|s__bromii	0.0167	44	45	0.0489
Carcinoma	p__Proteobacteria|c__Alphaproteobacteria|o__RF32	0.0062	30	45	0.0333
Carcinoma	p__Proteobacteria|c__Alphaproteobacteria|o__RF32|f__	0.0062	30	45	0.0333
Carcinoma	p__Proteobacteria|c__Alphaproteobacteria|o__RF32|f__|g__	0.0062	30	45	0.0333
Carcinoma	p__Proteobacteria|c__Alphaproteobacteria|o__RF32|f__|g__|s__	0.0062	30	45	0.0333
**Carcinoma**	**p__Tenericutes|c__Mollicutes|o__Anaeroplasmatales**	**0.0033**	**22**	**45**	**0.0244**
**Carcinoma**	**p__Tenericutes|c__Mollicutes|o__Anaeroplasmatales|f__Anaeroplasmataceae**	**0.0033**	**22**	**45**	**0.0244**
**Carcinoma**	**p__Tenericutes|c__Mollicutes|o__Anaeroplasmatales|f__Anaeroplasmataceae|g__Anaeroplasma**	**0.0033**	**22**	**45**	**0.0244**
**Carcinoma**	**p__Tenericutes|c__Mollicutes|o__Anaeroplasmatales|f__Anaeroplasmataceae|g__Anaeroplasma|s__**	**0.0033**	**22**	**45**	**0.0244**
	**Higher in the Walnut group**				
**Sarcoma**	**p__Firmicutes|c__Bacilli**	**0.0020**	**4**	**20**	**0.010**
Sarcoma	p__Firmicutes|c__Bacilli|o__Lactobacillales	0.0270	17	20	0.043
Sarcoma	p__Firmicutes|c__Bacilli|o__Lactobacillales|f__Lactobacillaceae	0.0308	18	20	0.045
Sarcoma	p__Firmicutes|c__Bacilli|o__Lactobacillales|f__Lactobacillaceae|g__Lactobacillus	0.0308	18	20	0.045
Sarcoma	p__Firmicutes|c__Bacilli|o__Lactobacillales|f__Lactobacillaceae|g__Lactobacillus|s__	0.0396	20	20	0.050
**Sarcoma**	**p__Firmicutes|c__Clostridia|o__Clostridiales|f__Ruminococcaceae|g__Ruminococcus|Other**	**0.0006**	**2**	**20**	**0.005**
Carcinoma	p__Bacteroidetes|c__Bacteroidia|o__Bacteroidales|f__	0.0009	11	45	0.0122
Carcinoma	p__Bacteroidetes|c__Bacteroidia|o__Bacteroidales|f__|g__	0.0009	11	45	0.0122
Carcinoma	p__Bacteroidetes|c__Bacteroidia|o__Bacteroidales|f__|g__|s__	0.0009	11	45	0.0122
Carcinoma	p__Bacteroidetes|c__Bacteroidia|o__Bacteroidales|f__Prevotellaceae|g__Prevotella|s__	0.0045	28	45	0.0311
**Carcinoma**	**p__Firmicutes|c__Bacilli**	**0.0183**	**45**	**45**	**0.0500**
Carcinoma	p__Firmicutes|c__Bacilli|o__Turicibacterales	0.0113	39	45	0.0433
Carcinoma	p__Firmicutes|c__Bacilli|o__Turicibacterales|f__Turicibacteraceae	0.0113	39	45	0.0433
Carcinoma	p__Firmicutes|c__Bacilli|o__Turicibacterales|f__Turicibacteraceae|g__Turicibacter	0.0113	39	45	0.0433
Carcinoma	p__Firmicutes|c__Bacilli|o__Turicibacterales|f__Turicibacteraceae|g__Turicibacter|s__	0.0113	39	45	0.0433
Carcinoma	p__Firmicutes|c__Clostridia|o__Clostridiales|f__Lachnospiraceae|g__CoprocoControlus|Other	0.0005	10	45	0.0111
Carcinoma	p__Firmicutes|c__Clostridia|o__Clostridiales|f__Lachnospiraceae|g__Moryella	0.0018	14	45	0.0156
Carcinoma	p__Firmicutes|c__Clostridia|o__Clostridiales|f__Lachnospiraceae|g__Moryella|s__	0.0018	14	45	0.0156
Carcinoma	p__Firmicutes|c__Clostridia|o__Clostridiales|f__Lachnospiraceae|g__Roseburia	0.0002	2	45	0.0022
Carcinoma	p__Firmicutes|c__Clostridia|o__Clostridiales|f__Lachnospiraceae|g__Roseburia|Other	0.0002	2	45	0.0022
Carcinoma	p__Firmicutes|c__Clostridia|o__Clostridiales|Other	0.0004	7	45	0.0078
Carcinoma	p__Firmicutes|c__Clostridia|o__Clostridiales|Other|Other	0.0004	7	45	0.0078
Carcinoma	p__Firmicutes|c__Clostridia|o__Clostridiales|Other|Other|Other	0.0004	7	45	0.0078
Carcinoma	p__Firmicutes|c__Clostridia|o__Clostridiales|f__Peptococcaceae|Other	0.0093	37	45	0.0411
Carcinoma	p__Firmicutes|c__Clostridia|o__Clostridiales|f__Peptococcaceae|Other|Other	0.0093	37	45	0.0411
Carcinoma	p__Firmicutes|c__Clostridia|o__Clostridiales|f__Ruminococcaceae|g__Oscillospira	0.0002	2	45	0.0022
Carcinoma	p__Firmicutes|c__Clostridia|o__Clostridiales|f__Ruminococcaceae|g__Oscillospira|Other	0.0003	6	45	0.0067
Carcinoma	p__Firmicutes|c__Clostridia|o__Clostridiales|f__Ruminococcaceae|g__Oscillospira|s__	0.0002	2	45	0.0022
**Carcinoma**	**p__Firmicutes|c__Clostridia|o__Clostridiales|f__Ruminococcaceae|g__Ruminococcus|Other**	**0.0001**	**1**	**45**	**0.0011**
Carcinoma	p__Proteobacteria|c__Alphaproteobacteria	0.005	29	45	0.0322
Carcinoma	p__Proteobacteria|c__Deltaproteobacteria|o__Desulfovibrionales|f__Desulfovibrionaceae|Other	0.004	26	45	0.0289
Carcinoma	p__Proteobacteria|c__Deltaproteobacteria|o__Desulfovibrionales|f__Desulfovibrionaceae|Other|Other	0.004	26	45	0.0289

^1^ Critical value for the Benjamini-Hochberg test as described by Benjamini and Hochberg [35]. Pr > ChiSq as determined by two-way ANOVA. False discovery rate 0.05. All species ranked above the highest *p*-value but less than CV are shown.

**Table A2:** Significantly different taxa based on treatment.

Tumor Type	Dependent Variable	Pr > ChiSq from Kruskal–Wallis Test	Rank	m = Number of Dependent Variables	CV for BH False Detect Rate ^1^	Group with the Greatest Amount	*p*-Value
**NTB-AL vs. NTB-PF**							
	p__Firmicutes						
Sarcoma	c__Clostridia|o__Clostridiales|f__Ruminococcaceae|g__	0.0004	1	16	0.003125	NTB-AL	0.0146
Sarcoma	c__Clostridia|o__Clostridiales|f__Ruminococcaceae|g__|s__	0.0004	1	16	0.003125	NTB-AL	0.0146
Sarcoma	c__Clostridia|o__Clostridiales|f__Veillonellaceae|g__Veillonella	0.0005	3	16	0.009375	NTB-AL	0.0057
Sarcoma	c__Clostridia|o__Clostridiales|f__Veillonellaceae|g__Veillonella|s__dispar	0.0005	3	16	0.009375	NTB-AL	0.0057
	p__Actinobacteria						
Carcinoma	c__Coriobacteriia	0.0013	7	41	0.0085	NTB-AL	0.0154
Carcinoma	c__Coriobacteriia|o__Coriobacteriales	0.0013	7	41	0.0085	NTB-AL	0.0154
Carcinoma	c__Coriobacteriia|o__Coriobacteriales|f__Coriobacteriaceae	0.0013	7	41	0.0085	NTB-AL	0.0154
	p__Bacteroidetes						
Carcinoma	c__Bacteroidia|o__Bacteroidales|f__Porphyromonadaceae|g__Parabacteroides	0.017	24	41	0.0293	NTB-AL	0.0216
Carcinoma	c__Bacteroidia|o__Bacteroidales|f__Porphyromonadaceae|g__Parabacteroides|s__	0.017	24	41	0.0293	NTB-AL	0.0216
Carcinoma	c__Bacteroidia|o__Bacteroidales|f__Rikenellaceae	0.0284	38	41	0.0463	NTB-AL	0.0406
	p__Firmicutes						
Carcinoma	c__Bacilli|o__Lactobacillales	0.0174	26	41	0.0317	NTB-AL	0.0406
	p__Proteobacteria						
Carcinoma	c__Gammaproteobacteria|o__Pasteurellales|f__Pasteurellaceae|g__Haemophilus	0.0102	22	41	0.0268	NTB-AL	0.0097
Carcinoma	c__Gammaproteobacteria|o__Pasteurellales|f__Pasteurellaceae|g__Haemophilus|s__parainfluenzae	0.0102	22	41	0.0268	NTB-AL	0.0097
**NTB-AL vs. TB**							
	p__Firmicutes						
Sarcoma	c__Clostridia|o__Clostridiales|f__Lachnospiraceae|g__Blautia	0.0019	6	16	0.01875	TB	0.0331
Sarcoma	c__Clostridia|o__Clostridiales|f__Lachnospiraceae|g__Blautia|Other	0.0009	5	16	0.015625	TB	0.0193
Sarcoma	c__Clostridia|o__Clostridiales|f__Ruminococcaceae|g__	0.0004	1	16	0.003125	TB	0.0427
Sarcoma	c__Clostridia|o__Clostridiales|f__Ruminococcaceae|g__|s__	0.0004	1	16	0.003125	TB	0.0427
	p__Elusimicrobia						
Carcinoma	p__Elusimicrobia	0.0199	27	41	0.0329	TB	0.039
Carcinoma	c__Elusimicrobia	0.0199	27	41	0.0329	TB	0.039
Carcinoma	c__Elusimicrobia|o__Elusimicrobiales	0.0199	27	41	0.0329	TB	0.039
Carcinoma	c__Elusimicrobia|o__Elusimicrobiales|f__Elusimicrobiaceae	0.0199	27	41	0.0329	TB	0.039
Carcinoma	c__Elusimicrobia|o__Elusimicrobiales|f__Elusimicrobiaceae|g__Elusimicrobium	0.0199	27	41	0.0329	TB	0.039
Carcinoma	c__Elusimicrobia|o__Elusimicrobiales|f__Elusimicrobiaceae|g__Elusimicrobium|s__	0.0199	27	41	0.0329	TB	0.039
	p__Firmicutes						
Carcinoma	c__Bacilli|o__Lactobacillales	0.0174	26	41	0.0317	NTB-AL	0.0463
Carcinoma	c__Bacilli|o__Lactobacillales|f__Lactobacillaceae|g__Lactobacillus|s__	0.0354	39	41	0.0476	NTB-AL	0.0463
	p__Proteobacteria						
Carcinoma	c__Betaproteobacteria	0.0015	11	41	0.0134	NTB-AL	0.0227
Carcinoma	c__Betaproteobacteria|o__Burkholderiales	0.0015	11	41	0.0134	NTB-AL	0.0227
Carcinoma	c__Betaproteobacteria|o__Burkholderiales|f__Alcaligenaceae	0.0015	11	41	0.0134	NTB-AL	0.0227
Carcinoma	c__Betaproteobacteria|o__Burkholderiales|f__Alcaligenaceae|g__Sutterella	0.0015	11	41	0.0134	NTB-AL	0.0227
Carcinoma	c__Betaproteobacteria|o__Burkholderiales|f__Alcaligenaceae|g__Sutterella|s__	0.0015	11	41	0.0134	NTB-AL	0.0227
	p__Tenericutes						
Carcinoma	p__Tenericutes	0.0014	10	41	0.0122	TB	0.0055
Carcinoma	c__Mollicutes	0.0273	33	41	0.0402	TB	0.0055
Carcinoma	c__Mollicutes|o__Anaeroplasmatales	0.0047	18	41	0.0220	TB	0.0273
Carcinoma	c__Mollicutes|o__Anaeroplasmatales|f__Anaeroplasmataceae	0.0047	18	41	0.0220	TB	0.0273
Carcinoma	c__Mollicutes|o__Anaeroplasmatales|f__Anaeroplasmataceae|g__Anaeroplasma	0.0047	18	41	0.0220	TB	0.0273
Carcinoma	c__Mollicutes|o__Anaeroplasmatales|f__Anaeroplasmataceae|g__Anaeroplasma|s__	0.0047	18	41	0.0220	TB	0.0273
**NTB-PF vs. TB**							
	p__Firmicutes						
Sarcoma	c__Bacilli|o__Lactobacillales	0.0211	12	16	0.0375	TB	0.0194
Sarcoma	c__Bacilli|o__Lactobacillales|f__Lactobacillaceae	0.0268	13	16	0.040625	TB	0.0249
Sarcoma	c__Bacilli|o__Lactobacillales|f__Lactobacillaceae|g__Lactobacillus	0.0268	13	16	0.040625	TB	0.0249
Sarcoma	c__Bacilli|o__Lactobacillales|f__Lactobacillaceae|g__Lactobacillus|s__	0.0268	13	16	0.040625	TB	0.0249
Sarcoma	c__Bacilli|o__Lactobacillales|f__Lactobacillaceae|g__Lactobacillus|s__reuteri	0.0297	16	16	0.05	TB	0.0249
Sarcoma	c__Bacilli|o__Lactobacillales|f__StreptocoControlaceae|g__StreptocoControlus	0.0063	8	16	0.025	TB	0.0087
Sarcoma	c__Bacilli|o__Lactobacillales|f__StreptocoControlaceae|g__StreptocoControlus|s__	0.0061	7	16	0.021875	TB	0.0087
Sarcoma	c__Clostridia|o__Clostridiales|f__Lachnospiraceae|g__Blautia	0.0019	6	16	0.01875	TB	0.0065
Sarcoma	c__Clostridia|o__Clostridiales|f__Lachnospiraceae|g__Blautia|Other	0.0009	5	16	0.015625	TB	0.0036
Sarcoma	c__Clostridia|o__Clostridiales|f__Ruminococcaceae|g__	0.0004	1	16	0.003125	TB	0.0027
Sarcoma	c__Clostridia|o__Clostridiales|f__Ruminococcaceae|g__|s__	0.0004	1	16	0.003125	TB	0.0027
Sarcoma	c__Clostridia|o__Clostridiales|f__Veillonellaceae|g__Veillonella	0.0005	3	16	0.009375	TB	0.0019
Sarcoma	c__Clostridia|o__Clostridiales|f__Veillonellaceae|g__Veillonella|s__dispar	0.0005	3	16	0.009375	TB	0.0019
Sarcoma	c__Erysipelotrichi	0.0085	9	16	0.028125	TB	0.0402
Sarcoma	c__Erysipelotrichi|o__Erysipelotrichales	0.0085	9	16	0.028125	TB	0.0402
Sarcoma	c__Erysipelotrichi|o__Erysipelotrichales|f__Erysipelotrichaceae	0.0085	9	16	0.028125	TB	0.0402
	p__Actinobacteria						
Carcinoma	c__Coriobacteriia	0.0013	7	41	0.0085	TB	0.0035
Carcinoma	c__Coriobacteriia|o__Coriobacteriales	0.0013	7	41	0.0085	TB	0.0035
Carcinoma	c__Coriobacteriia|o__Coriobacteriales|f__Coriobacteriaceae	0.0013	7	41	0.0085	TB	0.0035
Carcinoma	c__Bacteroidia|o__Bacteroidales|f__Rikenellaceae|g__AF12	0.0033	16	41	0.0195	TB	0.0028
Carcinoma	c__Bacteroidia|o__Bacteroidales|f__Rikenellaceae|g__AF12|s__	0.0033	16	41	0.0195	TB	0.0028
	p__Proteobacteria						
Carcinoma	c__Betaproteobacteria	0.0015	11	41	0.0134	NTB-PF	0.0044
Carcinoma	c__Betaproteobacteria|o__Burkholderiales	0.0015	11	41	0.0134	NTB-PF	0.0044
Carcinoma	c__Betaproteobacteria|o__Burkholderiales|f__Alcaligenaceae	0.0015	11	41	0.0134	NTB-PF	0.0044
Carcinoma	c__Betaproteobacteria|o__Burkholderiales|f__Alcaligenaceae|g__Sutterella	0.0015	11	41	0.0134	NTB-PF	0.0044
Carcinoma	c__Betaproteobacteria|o__Burkholderiales|f__Alcaligenaceae|g__Sutterella|s__	0.0015	11	41	0.0134	NTB-PF	0.0044
	p__Tenericutes						
Carcinoma	p__Tenericutes	0.0014	10	41	0.0122	TB	0.0035
Carcinoma	c__Mollicutes	0.0273	33	41	0.0402	TB	0.0028
Carcinoma	c__Mollicutes|o__RF39	0.0273	33	41	0.0402	TB	0.039
Carcinoma	c__Mollicutes|o__RF39|f__	0.0273	33	41	0.0402	TB	0.039
Carcinoma	c__Mollicutes|o__RF39|f__|g__	0.0273	33	41	0.0402	TB	0.039
Carcinoma	c__Mollicutes|o__RF39|f__|g__|s__	0.0273	33	41	0.0402	TB	0.039
Carcinoma	c__Mollicutes|o__Anaeroplasmatales	0.0047	18	41	0.0220	TB	0.0099
Carcinoma	c__Mollicutes|o__Anaeroplasmatales|f__Anaeroplasmataceae	0.0047	18	41	0.0220	TB	0.0099
Carcinoma	c__Mollicutes|o__Anaeroplasmatales|f__Anaeroplasmataceae|g__Anaeroplasma	0.0047	18	41	0.0220	TB	0.0099
Carcinoma	c__Mollicutes|o__Anaeroplasmatales|f__Anaeroplasmataceae|g__Anaeroplasma|s__	0.0047	18	41	0.0220	TB	0.0099

^1^ Critical value for the Benjamini–Hochberg test as described by Benjamini and Hochberg [35]. Pr > ChiSq as determined by two-way ANOVA. False discovery rate is 0.05. All species ranked above the highest *p*-value but less than CV are shown.
